# Topology Optimization of Piezoelectric Energy Harvesters for Enhanced Open-Circuit Voltage Subjected to Harmonic Excitations

**DOI:** 10.3390/ma15134423

**Published:** 2022-06-22

**Authors:** Meng He, Mu He, Xiaopeng Zhang, Liang Xia

**Affiliations:** 1 The State Key Laboratory of Digital Manufacturing Equipment and Technology, Huazhong University of Science and Technology, 1037 Luoyu Road, Wuhan 430074, China; hemeng@hust.edu.cn (M.H.); muhe@hust.edu.cn (M.H.); 2 The State Key Laboratory of Structural Analysis for Industrial Equipment, Dalian University of Technology, 2 Linggong Road, Dalian 116023, China; zhangxiaopeng@dlut.edu.cn

**Keywords:** topology optimization, piezoelectric energy harvesters, open-circuit voltage, energy conversion efficiency, voltage cancellation

## Abstract

Energy harvesting devices made of piezoelectric material are highly anticipated energy sources for power wireless sensors. Tremendous efforts have been made to improve the performance of piezoelectric energy harvesters (PEHs). Noticeably, topology optimization has shown an attractive potential to design PEHs with enhanced energy conversion efficiency. In this work, an alternative yet more practical design objective was considered, where the open-circuit voltage of PEHs is enhanced by topologically optimizing the through-thickness piezoelectric material distribution of plate-type PEHs subjected to harmonic excitations. Compared to the conventional efficiency-enhanced designs, the open-circuit voltage of PEHs can be evidently enhanced by the proposed method while with negligible sacrifice on the energy conversion efficiency. Numerical investigations show that the voltage cancellation effect due to inconsistent voltage phases can be effectively ameliorated by optimally distributed piezoelectric materials.

## 1. Introduction

Energy harvesting devices made of piezoelectric material are highly anticipated energy sources for power wireless sensors [1,2]. Tremendous efforts have been made to improve the performance of piezoelectric energy harvesters (PEHs). According to the literature, PEHs were mainly designed for energy conversion efficiency [3,4], output power [5,6] and bandwidth [7,8]. From a practical point of view, the electric power generated by PEHs is of the most interest. Therefore, numerous researches focused on designing PEHs to achieve higher output power with broader operating bandwidth. To this end, various shapes were proposed for the piezoelectric patch layer of cantilever-type PEHs, such as rectangles [9], triangles [10] and trapezoids [11]. It was shown that the voltage frequency response and harvested power of PEHs could be enhanced by modifying the in-plane geometry or introducing through-thickness geometrical features within the piezoelectric layer, such as cross-section [12], cavities [13] and gaps [14] of varying sizes. Though the voltage or output power of PEHs can be increased to a certain extent by these designs, the overall efficiency is maintained at a low level for the confined design space.

Noticeably, topology optimization [15] has shown an attractive potential to design PEHs with enhanced energy conversion efficiency [16,17]. To our best knowledge, Zheng et al. [18] are the first to introduce topology optimization for PEHs, considering the energy conversion efficiency as the design objective. Chen et al. [19] proposed to topologically design PEHs under harmonic loads with the adoption of level-set methods [20,21]. Kim et al. [22] demonstrated that the intrinsic and objective-dependent conditions should be satisfied simultaneously to achieve stable convergence of the topological evolution of PEHs when considering three penalty exponents. Vatanabe et al. [23] proposed to design functionally graded piezocomposite materials using topology optimization for enhanced electromechanical coupling coefficient. Almeida and Pavanello [24] topologically designed the through-thickness geometrical features of a bimorph harvester using the bi-directional evolutionary structural optimization method [25]. He et al. [26] proposed a multi-material topology optimization method to enhance the energy conversion efficiency of PEHs with simultaneous distribution of the elastic, piezoelectric and void materials. Previous investigations showed that the amount of piezoelectric material embedded in structures is positively correlated to the energy conversion efficiency [22,27]. The PEH energy conversion efficiency enhancement by means of topology optimization was comprehensively reviewed recently by Amlashi et al. [28].

It should be noted that enhancing the energy conversion efficiency of PEHs does not necessarily result in a high output voltage or power, which is, however, of more importance in practical uses of miniature sensors such as ambient light, temperature and pressure sensors. It was shown that the output voltage or power can be enhanced through the designs of geometry, tip mass position, piezoelectric material layout and electrodes [29]. Rupp et al. [30] were the first to pave the way for topology optimization of PEHs with respect to the power considering harmonic excitations. It was shown that in order to enhance the output power, the piezoelectric patch needs to be topologically optimized to alter the structural modes such that the structure is simultaneously tuned to the driving frequency and prevents charge cancellation. Following this framework, Lee and Youn [31] designed a piezoelectric skin patched to an outdoor condensing unit to harvest vibration energy and experimentally validated the performance; Noh and Yoon [27] demonstrated the effects of penalization of the piezoelectric material interpolation model in topology optimization on the harvesting efficiency. Notice that the aforementioned designs for output power mainly focused on the in-plane material distribution of the piezoelectric patch, while the designs of the through-thickness geometrical features of plate-type PEHs were limited except for the works reported by Vatanabe et al. [32] and Wein et al. [33].

This work followed the same framework developed by Rupp et al. [30] and focused on the design of the through-thickness piezoelectric material distribution of plate-type PEHs subjected to harmonic excitations. The open-circuit voltage was considered as the design objective for simplicity consideration, which is equivalent to a power design assuming an infinitely large resistor connected. Meanwhile, the energy conversion efficiency was also considered for design. The results were compared to those obtained from open-circuit voltage designs. The innovation of this paper was to achieve the performance optimization of piezoelectric materials along the thickness direction at both low and high frequencies. The remainder of this paper is organized as follows. Section 2 briefly reviews the finite element formulations of piezoelectric structures. Section 3 establishes the topology optimization model and considers, respectively, the open-circuit voltage and the energy conversion efficiency as the design objective. The analytical sensitivities derived from the two design objectives were also demonstrated. Section 4 validates the method through a series of designs of a benchmark clamped–clamped piezoelectric plate. The conclusion is drawn in Section 5.

## 2. Finite Element Analysis of Piezoelectric Structures

The piezoelectric material can convert mechanical into electrical energy and vice versa. The linearly coupled mechanical and electrical constitutive behavior of piezoelectric material can be written in the following stress-charge form as [34]:

(1) $$\;\{\begin{array}{l} \sigma =C^{E}\varepsilon -eE\\ D=e^{T}\varepsilon +\kappa^{S}E \end{array}$$

where σ and ε are the mechanical stress and strain tensors, respectively, and D is the electric displacement vector. $C^{E}$ and $\kappa^{S}$ are the elasticity and dielectric permittivity tensors at constant electric field E and constant mechanical strain ε, correspondingly. e is the piezoelectric coefficient tensor, and the superscript T denotes matrix transposition. The generalized matrix dimension of $C^{E}$ and $\kappa^{S}$ are 6 by 6 and 3 by 3 symmetric matrices, respectively, and e is a 6 by 3 matrix. As the plane strain assumption is adopted, the dimensions of the matrices $C^{E}$ and $\kappa^{S}$ are thus 3 by 3 and 2 by 2, respectively, and the e is a 3 by 2 matrix.

**Figure 1:**
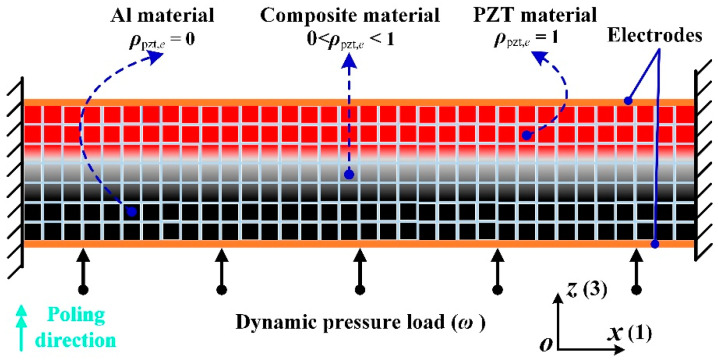
Schematic representation of a clamped–clamped piezoelectric energy harvesting structure.

Due to the brittleness of piezoceramic, the piezoelectric energy harvesting structure consists of a piezoelectric domain and an elastic (aluminum or copper) support layer. Figure 1 shows the schematic representation of the modeled piezoelectric energy harvesting structure under the plane strain assumption. The solid arrows with point represent a dynamic pressure load acting on the bottom surface. The direction of PZT material is polarized along the *z*(3) direction, which is indicated by the solid double arrow.The mechanical properties (stiffness) of these physical electrodes can be neglected, while for the electric property, the top and bottom electrodes represent two equipotential surfaces in the model.

As shown in Figure 1, the design domain is assumed to be discretized into *N* square finite elements, and each element is associated with the continuously defined topology variable $\rho_{\mathrm{pzt},e}$, denoting the existence of piezoelectric material:

(2) $$0\leq \rho_{\mathrm{pzt},e}\leq 1\;\mathrm{with}\;e=1,\;2,\ldots ,\;N.$$

Following the piezoelectric material with penalization (PEMAP) model [35], which is an extension of the well-known solid isotropic material with penalization (SIMP) model [15], the topology variable is linked to material constitutive parameters in the following form:

(3) $$m\left(\rho_{\mathrm{pzt},e}\right)=\rho_{\mathrm{pzt},e}m_{\mathrm{pzt},e}+\left(1-\rho_{\mathrm{pzt},e}\right)m_{\mathrm{nonpzt}}$$

(4) $$\;C\left(\rho_{\mathrm{pzt},e}\right)=\rho_{\mathrm{pzt},e}^{p_{uu}}C_{\mathrm{pzt}}+\left(1-\rho_{\mathrm{pzt},e}^{p_{uu}}\right)C_{\mathrm{nonpzt}}$$

(5) $$\;e\left(\rho_{\mathrm{pzt},e}\right)=\rho_{\mathrm{pzt},e}^{p_{u\phi }}e_{\mathrm{pzt}}\;$$

(6) $$\;\kappa \left(\rho_{\mathrm{pzt},e}\right)=\rho_{\mathrm{pzt},e}^{p_{\phi \phi }}\kappa_{\mathrm{pzt}}\;$$

where {$m_{\mathrm{pzt}}$,$C_{\mathrm{pzt}}$} and {$m_{\mathrm{nonpzt}}$,$C_{\mathrm{nonpzt}}$} are, respectively, the mass and elastic tensors of piezoelectric and non-piezoelectric materials; $e_{\mathrm{pzt}}$ and $\kappa_{\mathrm{pzt}}$ are the piezoelectric coupling and dielectric coefficients, respectively; $p_{uu}$, $p_{u\phi }$ and $p_{\phi \phi }$ are penalization coefficients for the elastic, piezoelectric coupling and dielectric properties, respectively. According to the above material interpolation model, the piezoelectric material is obtained with $\rho_{\mathrm{pzt},e}=1$; elastic material (aluminum) is obtained with $\rho_{\mathrm{pzt},e}=0$. Note that intermediate values ($0<\rho_{\mathrm{pzt},e}<1$) represent composite material elements without physical meanings. These artificial densities should be penalized in a manner analogous to other continuous optimization approximations of 0–1 problems [15]. Details about the setting of the penalization coefficients are provided in Appendix A.

By using Hamilton’s variational principle and neglecting the damping effect, the detailed finite element derivation and calculation of the piezoelectric element matrix are introduced in reference [34]. It can be seen in Figure 1 that each square element has four nodes, and each node has three degrees of freedom (DOFs) (two mechanical DOFs regarding the displacements in *x* and *z* directions and one electric potential DOF). The finite element formulation of piezoelectric structures subjected to harmonic excitations can be written as follows:

(7) $$\;\left[\begin{array}{cc} M & 0\\ 0 & 0 \end{array}\right]\left\{\begin{array}{c} {\ddot{U}}_{t}\\ {\ddot{\Phi }}_{t} \end{array}\right\}+\left[\begin{array}{cc} K_{uu} & K_{u\phi }\\ K_{\phi u} & -K_{\phi \phi } \end{array}\right]\left\{\begin{array}{c} U_{t}\\ \Phi_{t} \end{array}\right\}=\left\{\begin{array}{c} F_{t}\\ Q_{t} \end{array}\right\}$$

where $F_{t}$ and $Q_{t}$ are the time-harmonic excitations of mechanical force and electrical charge with $\left\{F_{t}\;Q_{t}\right\}=\left\{F\;Q\right\}e^{i\omega t}$, $U_{t}\left(m\times 1\right)$ and $\Phi_{t}\left(n\times 1\right)$ are the time-varying responses of displacement and electrical potential with $\left\{U_{t}\;\Phi_{t}\right\}=\left\{U\;\Phi \right\}e^{i\omega t}$. F(m×1), Q(n×1), U(m×1) and Φ(n×1) are the amplitudes of harmonic force, electrical charge, displacement and potential, respectively. M(m×m), $K_{uu}\left(m\times m\right)$, $K_{u\phi }\left(m\times n\right)$ and $K_{\phi \phi }\left(n\times n\right)$ are, respectively, the global mass, stiffness, piezoelectric coupling and dielectric matrices. The external charge Q is set to zero for energy harvesting problems. The above harmonic analysis formulation can be further derived to the following simplified form [34]:

(8) $$\left[\begin{array}{cc} K_{uu}-M\omega^{2} & K_{u\phi }\\ K_{\phi u} & -K_{\phi \phi } \end{array}\right]\left\{\begin{array}{c} U\\ \Phi \end{array}\right\}\begin{array}{c} =\left[\begin{array}{cc} {\bar{K}}_{uu} & K_{u\phi }\\ K_{\phi u} & -K_{\phi \phi } \end{array}\right]\left\{\begin{array}{c} U\\ \Phi \end{array}\right\}=\left\{\begin{array}{c} F\\ Q \end{array}\right\} \end{array}$$

where ω is the imposed excitation frequencies (f=ω/2π). The piezoelectric plate for energy harvesting is commonly sandwiched between two silver electrode layers. In practice, the electrode layers are assumed to be zero, and their effect is equivalently modeled by imposing equipotential boundary conditions [28]. The potentials of the nodes on the electrode layers are gathered from the global potential vector by using a Boolean matrix, and their values are enforced to be the same. This study focused on the application of topology optimization to optimize PEHs by developing an in-house FE code under dynamic loads.

## 3. Optimization Model and Sensitivity Analysis

The design objectives were considered, respectively, in this work, namely, the energy conversion efficiency and the open-circuit voltage. The energy conversion efficiency is defined as the ratio of the generated electric energy to the external mechanical work [26]:

(9) $$\;\eta_{\mathrm{energy}}=\frac{\Pi_{E}}{\Pi_{S}+\Pi_{E}}$$

Here, $\Pi_{S}=\left(1/4\right)U^{T}K_{uu}U$ and $\Pi_{E}=\left(1/4\right)\Phi^{T}K_{\phi \phi }\Phi$ are the elastic and dielectric energies under harmonic analysis. Note that harmonic stress and strain components are generally not in phase with each other, so the two energies are estimated as the real parts of their cycle averaged values.

The open-circuit voltage is defined as the electric potential difference between the top and bottom electrode layers of the piezoelectric patch, as shown in Figure 1. Since equipotential boundary conditions are imposed at the two layers, the potential of an arbitrarily prescribed node on the top layer can be adopted for denoting the open-circuit voltage:

(10) $$\;\eta_{\mathrm{voltage}}=L_{\mathrm{dummy}}^{T}\Phi_{\mathrm{oc}}$$

where $\Phi_{\mathrm{oc}}$ is the global electric potential vector, $L_{\mathrm{dummy}}$ is a selecting vector in which the component corresponding to the prescribed node is set to one while the others are zero. In the following analysis, the open-circuit voltage is measured without connecting to an external harvesting circuit, i.e., an infinite external resistance is assumed.

By considering the above two properties as design objectives, the mathematical model for topology optimization of piezoelectric materials subjected to harmonic excitations is formulated as follows:
find:  $\rho_{\mathrm{pzt}}$                  (11)max:  $\eta_{\mathrm{energy}}\;\mathrm{or}\;\eta_{\mathrm{voltage}}$s.t.  ${\bar{K}}_{uu}U+K_{u\phi }\Phi =F$ $K_{\phi u}U-K_{\phi \phi }\Phi =Q$ $V\left(\rho_{\mathrm{pzt}}\right)/V_{0}\leq \bar{V}$ $0\leq \rho_{\mathrm{pzt}}\leq 1$
where $V\left(\rho_{\mathrm{pzt}}\right)$ and $V_{0}$ are, respectively, the volumes of piezoelectric material and design domain, and $\bar{V}$ is the prescribed volume fraction.

In order to favor gradient-based mathematical programming algorithms [36,37], sensitivities of the two design objectives with respect to topology variables are derived using the adjoint method. The sensitivity of energy conversion efficiency w.r.t. $\rho_{\mathrm{pzt},e}$ is derived as:

(12) $$\;\begin{array}{c} \frac{\partial \eta_{\mathrm{energy}}}{\partial \rho_{\mathrm{pzt},e}}=\frac{\partial \left(U^{T}K_{uu}U\right)/\partial \rho_{\mathrm{pzt},e}}{\left(\Phi^{T}K_{\phi \phi }\Phi \right)}-\frac{\left(\partial \left(\Phi^{T}K_{\phi \phi }\Phi \right)/\partial \rho_{\mathrm{pzt},e}\right)U^{T}K_{uu}U}{{\left(\Phi^{T}K_{\phi \phi }\Phi \right)}^{2}} \end{array}$$

The derivatives of the elastic and dielectric energies w.r.t. $\rho_{\mathrm{pzt},e}$ can be obtained as [26]:

(13) $$\{\begin{array}{l} \frac{\partial \left(U^{T}K_{uu}U\right)}{\partial \rho_{\mathrm{pzt},e}}=\left(U^{T}+\lambda_{1}^{T}\right)\frac{\partial K_{uu}}{\partial \rho_{\mathrm{pzt},e}}U+\lambda_{1}^{T}\frac{\partial K_{u\phi }}{\partial \rho_{\mathrm{pzt},e}}\Phi \\ +\mu_{1}^{T}\frac{\partial K_{\phi u}}{\partial \rho_{\mathrm{pzt},e}}U-\mu_{1}^{T}\frac{\partial K_{\phi \phi }}{\partial \rho_{\mathrm{pzt},e}}\Phi -\lambda_{1}^{T}\frac{\partial M\omega^{2}}{\partial \rho_{\mathrm{pzt},e}}U\\ \frac{\partial \left(\Phi^{T}K_{\phi \phi }\Phi \right)}{\partial \rho_{\mathrm{pzt},e}}=\lambda_{2}^{T}\frac{\partial K_{uu}}{\partial \rho_{\mathrm{pzt},e}}U+\lambda_{2}^{T}\frac{\partial K_{u\phi }}{\partial \rho_{\mathrm{pzt},e}}\Phi \\ +\mu_{2}^{T}\frac{\partial K_{\phi u}}{\partial \rho_{\mathrm{pzt},e}}U-\left(\Phi^{T}-\mu_{2}^{T}\right)\frac{\partial K_{\phi \phi }}{\partial \rho_{\mathrm{pzt},e}}\Phi -\lambda_{2}^{T}\frac{\partial M\omega^{2}}{\partial \rho_{\mathrm{pzt},e}}U \end{array}$$

where $\lambda_{1}$, $\mu_{1}$, $\lambda_{2}$ and $\mu_{2}$ are solutions of the following two adjoint problems:

(14) $$\;\left[\begin{array}{cc} {\bar{K}}_{uu} & K_{u\phi }\\ K_{\phi u} & -K_{\phi \phi } \end{array}\right]\left\{\begin{array}{c} \lambda_{1}\\ \mu_{1} \end{array}\right\}=\left\{\begin{array}{c} -2K_{uu}\\ 0 \end{array}\right\}\;\mathrm{and}\;\left[\begin{array}{cc} {\bar{K}}_{uu} & K_{u\phi }\\ K_{\phi u} & -K_{\phi \phi } \end{array}\right]\left\{\begin{array}{c} \lambda_{2}\\ \mu_{2} \end{array}\right\}=\left\{\begin{array}{c} 0\\ -2K_{\phi \phi } \end{array}\right\}$$

The derivatives of the constitutive matrices w.r.t. $\rho_{\mathrm{pzt}}$ in Equation (13) can be straightforwardly derived according to the defined PEMAP material interpolation model.

In order to simplify the mathematical minimization problem, the design objective of open-circuit voltage can be equivalently reformulated in the following form:

(15) $$\eta_{\mathrm{voltage}}=-L_{\mathrm{dummy}}^{T}\Phi_{\mathrm{oc}}+\lambda_{3}^{T}\left({\bar{K}}_{uu}U+K_{u\phi }\Phi -F\right)+\mu_{3}^{T}\left(K_{u\phi }^{T}U-K_{\phi \phi }\Phi -Q\right)$$

Similarly, the sensitivity of open-circuit voltage w.r.t. $\rho_{\mathrm{pzt},e}$ based on the adjoint variable method is derived as:

(16) $$\begin{array}{c} \frac{\partial \eta_{\mathrm{voltage}}}{\partial \rho_{\mathrm{pzt},e}}=\frac{\partial \left(-L_{\mathrm{dummy}}^{T}\Phi_{\mathrm{oc}}\;+\;\lambda_{3}^{T}\left({\bar{K}}_{uu}U\;+\;K_{u\phi }\Phi -F\right)\right)}{\partial \rho_{\mathrm{pzt},e}}\;+\;\frac{\partial \left(\begin{array}{c} \mu_{3}^{T}\left(K_{u\phi }^{T}U\;-\;K_{\phi \phi }\Phi \;-\;Q\right) \end{array}\right)}{\partial \rho_{\mathrm{pzt},e}} \end{array}$$

Considering that $(\partial F/\partial \rho_{\mathrm{pzt},e})=0$ and Q=0, the sensitivity equation can be further reduced by algebraic operations:

(17) $$\begin{array}{l} \frac{\partial \eta_{\mathrm{voltage}}}{\partial \rho_{\mathrm{pzt},e}}=\left(\lambda_{3}^{T}{\bar{K}}_{uu}+\mu_{3}^{T}K_{u\phi }^{T}\right)\frac{\partial U}{\partial \rho_{\mathrm{pzt},e}}+\left(-L_{\mathrm{dummy}}^{T}+\lambda_{3}^{T}K_{u\phi }-\mu_{3}^{T}K_{\phi \phi }\right)\frac{\partial \Phi }{\partial \rho_{\mathrm{pzt},e}}\\ \;+\lambda_{3}^{T}\frac{\partial {\bar{K}}_{uu}}{\partial \rho_{\mathrm{pzt},e}}U+\lambda_{3}^{T}\frac{\partial K_{u\phi }}{\partial \rho_{\mathrm{pzt},e}}\Phi +\mu_{3}^{T}\frac{\partial K_{u\phi }^{T}}{\partial \rho_{\mathrm{pzt},e}}U-\mu_{3}^{T}\frac{\partial K_{\phi \phi }}{\partial \rho_{\mathrm{pzt},e}}\Phi \end{array}$$

In which the derivatives of the two-state variables {$\partial U/\partial \rho_{\mathrm{pzt},e}$, $\partial \Phi /\partial \rho_{\mathrm{pzt},e}$} can be eliminated with the solutions of the following adjoint problem:

(18) $$\left[\begin{array}{cc} {\bar{K}}_{uu} & K_{u\phi }\\ K_{\phi u} & -K_{\phi \phi } \end{array}\right]\left\{\begin{array}{c} \lambda_{3}\\ \mu_{3} \end{array}\right\}=\left\{\begin{array}{c} 0\\ L_{\mathrm{dummy}}^{T} \end{array}\right\}$$

Additionally, the final sensitivity estimation form is derived as:

(19) $$\begin{array}{c} \frac{\partial \eta_{\mathrm{voltage}}}{\partial \rho_{\mathrm{pzt},e}}=\lambda_{3}^{T}\frac{\partial K_{uu}}{\partial \rho_{\mathrm{pzt},e}}U+\lambda_{3}^{T}\frac{\partial K_{u\phi }}{\partial \rho_{\mathrm{pzt},e}}\Phi +\mu_{3}^{T}\frac{\partial K_{u\phi }^{T}}{\partial \rho_{\mathrm{pzt},e}}U-\mu_{3}^{T}\frac{\partial K_{\phi \phi }}{\partial \rho_{\mathrm{pzt},e}}\Phi -\lambda_{3}^{T}\frac{\partial M\omega^{2}}{\partial \rho_{\mathrm{pzt},e}}U. \end{array}$$

To subsequently calculate Equation (19), all the derivative terms can be obtained from the interpolation scheme in Equations (5)–(8). Herein, we did not develop it.

## 4. Numerical Examples

**Figure 2:**
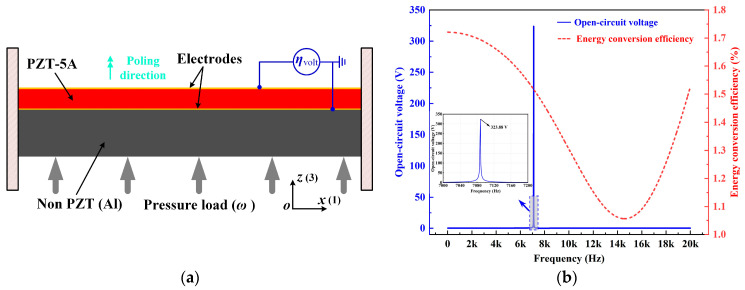
Schematic diagram and frequency response curve of PEH. (**a**) Schematic cross-section of a harmonically pressured clamped–clamped piezoelectric plate; (**b**) frequency responses of the open-circuit voltage and the energy conversion efficiency.

As is shown in Figure 2a, a specified unimorph piezoelectric clamped–clamped plate structure without intermediate variables of Figure 1 is considered for the original design. The dimensions of the cross-section are 0.1 m × 0.02 m, and the height of the PZT-5A layer is 0.006 m. The design domain is uniformly discretized into 100 × 20 bilinear square elements with the plane strain assumption. The material properties of PZT-5A are *c*_11_ = 120.3 GPa, *c*_12_ = 75.2 GPa, *c*_13_ = 75.1 GPa, *c*_33_ = 110.8 GPa, *c*_44_ = 21.1 GPa, *c*_66_ = 22.6 GPa, *e*_31_ = −5.4 C/m^2^, *e*_33_ = 15.8 C/m^2^, *e*_15_ = 12.3 C/m^2^, *κ*_11_ = 919.1, *κ*_33_ = 826.6, *κ*_0_ = 8.55 × 10^−12^ F/m, and the density is 7500 kg/m^3^. The Young’s modulus, density and Poisson’s ratio of the aluminum are 71 GPa, 2700 kg/m^3^ and 0.33, respectively. A harmonic pressure load with an amplitude of 10 kPa was applied on the bottom surface. For the optimization process, we used the in-house MATLAB code that considers the dynamic pressure loads. The developed FE code was then verified using commercial software COMSOL. The parameters of the applied piezoelectric material PZT-5A are given as follows:

- Elastic matrix: $C_{2D}^{E}=\left[\begin{array}{ccc} 120.3 & 75.1 & 0\\ 75.1 & 110.8 & 0\\ 0 & 0 & 21.1 \end{array}\right]$ GPa;
- Piezoelectric coupling matrix: $e_{2D}^{T}=\left[\begin{array}{c} \begin{array}{ccc} 0 & 0 & 12.3 \end{array}\\ \begin{array}{ccc} -5.4 & 15.8 & 0 \end{array} \end{array}\right]$ C/m^2^;
- Dielectric matrix: $\kappa_{2D}^{s}=\left[\begin{array}{cc} 919.1 & 0\\ 0 & 826.6 \end{array}\right]\times 8.55\times 10^{-12}$ F/m.

The frequency responses of the original design are also shown in Figure 2b. The energy conversion efficiency varies along with the excitation frequency while holding a constant low-level value. The open-circuit voltage is also very small within the frequency range except for an abrupt jump at the resonance.

**Figure 3:**
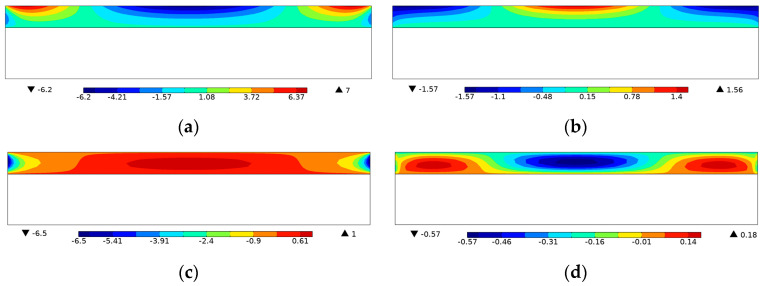
Influence of the equipotential boundary conditions on voltage contour. (**a**) Without equipotential constraint *f* = 4 kHz; (**b**) without equipotential constraint *f* = 16 kHz; (**c**) with equipotential constraint *f* = 4 kHz; (**d**) with equipotential constraint *f* = 16 kHz.

In order to simulate the effect of the two electrode layers in Figure 2a, equipotential boundary conditions were imposed, and their influence on the energy harvesting performance was demonstrated in Figure 3 and Table 1. It can be observed that the potential distribution within the piezoelectric layer was changed as a result of the equipotential boundary conditions. Notice that a piezoelectric patch without electrodes is practically useless as the voltage varies along the length (*x*) direction, and thus there exists no specific output open-circuit voltage or power. Therefore, the attachment of electrode layers is necessary, and it inevitably brings the issue of charge or voltage cancellation [38,39], resulting in the sacrifice of the energy harvesting performance. In the following, the PZT-5A material distribution was topologically optimized for energy conversion efficiency and open-circuit voltage in Section 4.1 and Section 4.2, respectively. The filter radius was set as two times the element length. The volume fraction of PZT-5A was constrained below 30%. The method of moving asymptotes (MMA, [40]) was adopted as the optimizer. Additionally, the post-processing scheme with a threshold was adopted to eliminate intermediate densities [41].

### 4.1. Design for Enhanced Energy Conversion Efficiency

**Figure 4:**
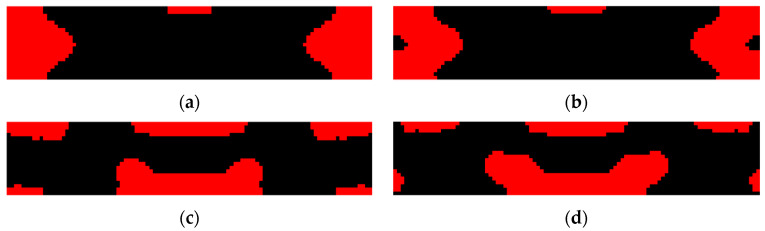
Energy conversion efficiency enhanced designs for variant excitation frequencies. (**a**) *f* = 4 kHz; (**b**) *f* = 8 kHz; (**c**) *f* = 12 kHz; (**d**) *f* = 16 kHz.

**Figure 5:**
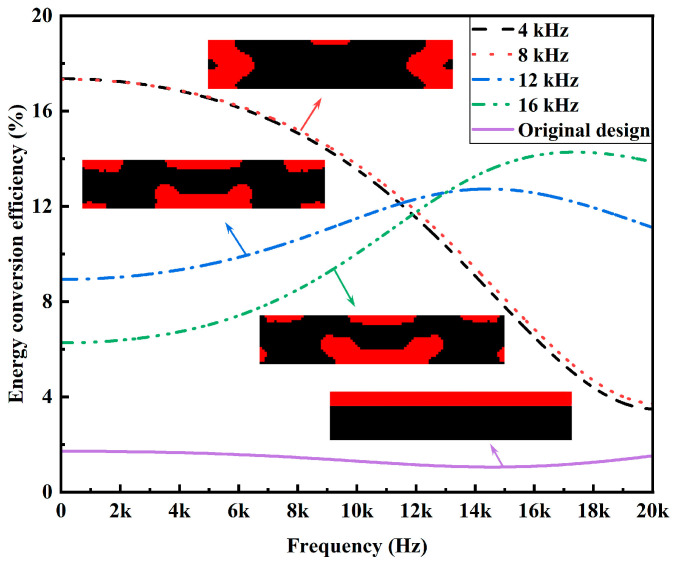
Frequency responses of the energy conversion efficiencies of the enhanced designs.

The energy conversion efficiency enhanced designs obtained by topology optimization for variant excitation frequencies are shown in Figure 4 and Table 2. For each of the excitation frequencies, the designed PZT-5A material distribution enhances an average 10 times the efficiency than that of the original design with top-layered PZT-5A material. Meanwhile, it can also be observed that the designed PZT-5A material distribution is highly dependent on the excitation frequency. Generally speaking, PZT-5A material is preferentially distributed in highly strained regions so as to generate more dielectric energy. This explains why PZT-5A materials are distributed around the two clamped ends for low-frequency excitations while they are distributed towards the center for high-frequency excitations. Frequency responses of the energy conversion efficiencies of the enhanced designs are shown in Figure 5. The frequency responses of the designs of Figure 4a,b are almost identical, which can actually be anticipated from their similar PZT-5A distribution. The designs of Figure 4c,d evidently outperforms the designs of Figure 4a,b in terms of the efficiency for high excitation frequencies, while the opposite for low excitation frequencies.

**Figure 6:**
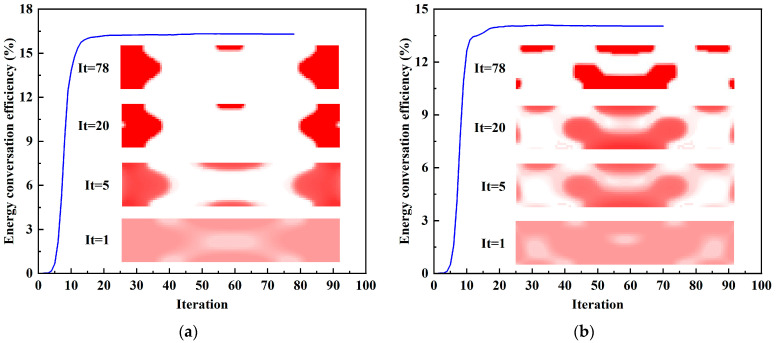
Iterative history and topological evolution for energy conversion efficiency designs. (**a**) *f* = 4 kHz; (**b**) *f* = 16 kHz.

The iterative history and topological evolution at 4 kHz and 16 kHz during the optimization steps are shown in Figure 6. As the iteration progresses, the topological configuration becomes clearer. Meanwhile, the energy conversion efficiency gradually increases and finally tends to be stable.

**Figure 7:**
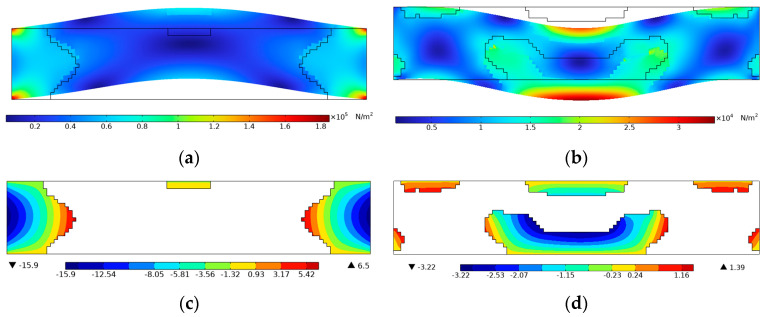
Demonstration of stress mode shape and voltage of Figure 4a,d. (**a**) Von Mises stress contour of Figure 4a, *f* = 4 kHz; (**b**) Von Mises stress contour of Figure 4d, *f* = 16 kHz; (**c**) Voltage contour of Figure 4a, *f* = 4 kHz; (**d**) Voltage contour of Figure 4d, *f* = 16 kHz.

For further demonstration, the Von Mises stress and voltage contours of the efficiency-enhanced designs of Figure 4a,d are shown in Figure 7. From the vibration mode shape of the optimized designs in Figure 7a,b, it can be seen that the PZT-5A material is preferentially distributed at high stressed regions for efficiency enhancement purposes. On the contrary, the voltage contours in Figure 7c,d show that positive and negative voltages are canceled along the length direction of the top electrode layer, resulting in open-circuit voltages of low magnitude. In order to be consistent with the settings of the original design in Figure 2, equipotential boundary conditions are imposed on the top layer to simulate the electrode layer, and the bottom layer is grounded, as shown in Figure 7c,d. The same electrical boundary conditions are imposed in the following designs for open-circuit voltage.

### 4.2. Design for Enhanced Open-Circuit Voltage

**Figure 8:**
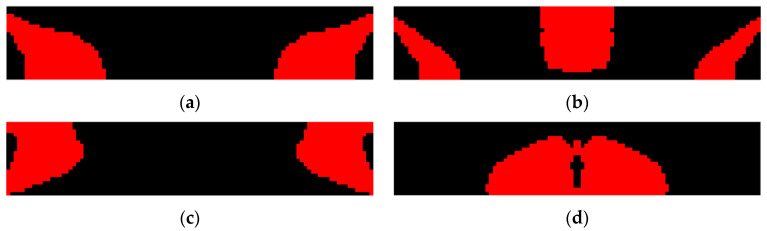
Open-circuit voltage enhanced designs for variant excitation frequencies. (**a**) *f* = 4 kHz; (**b**) *f* = 6 kHz; (**c**) *f* = 10 kHz; (**d**) *f* = 16 kHz.

Topology optimization is further conducted considering the open-circuit voltage as the design objective to maximize, and the results are shown in Figure 8. Unlike the previous efficiency enhanced designs, open-circuit voltage enhanced designs do not exhibit an obvious evolution tendency of PZT-5A material distribution on the excitation frequency. Table 3 shows the optimization results of the open-circuit voltage enhanced designs at variant excitation frequencies. It was noticed that in order to enhance the open-circuit voltage, PZT-5A material is distributed such that the first or second structural eigenfrequency is tuned to approach the excitation frequency for this design case. Convergence challenges encountered near the resonance for the excitation frequency ranged from 6.5 kHz to 9.5 kHz because the material distribution considerably changes the structural eigenfrequencies and vice versa.

**Figure 9:**
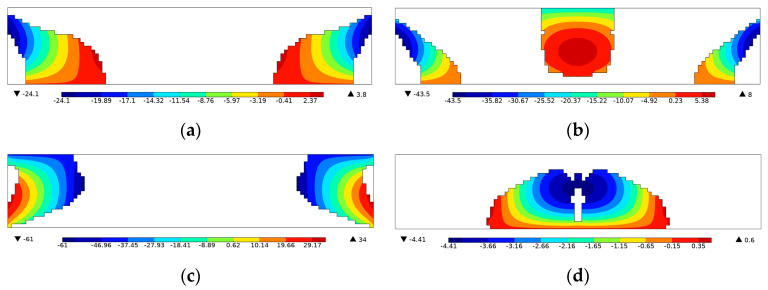
Voltage distribution of the open-circuit voltage enhanced designs in Figure 8. (**a**) Voltage contour of Figure 8a, *f* = 4 kHz; (**b**) Voltage contour of Figure 8b, *f* = 6 kHz; (**c**) Voltage contour of Figure 8c, *f* = 10 kHz; (**d**) Voltage contour of Figure 8d, *f* = 16 kHz.

Figure 9 shows the voltage distribution of the open-circuit voltage enhanced designs. Unlike the previous efficiency enhanced designs where the voltage cancellation happens along the length direction, the issue is evidently ameliorated in the voltage enhanced designs that the voltage monotonically increases through the thickness direction, resulting in open-circuit voltages of high magnitude.

**Figure 10:**
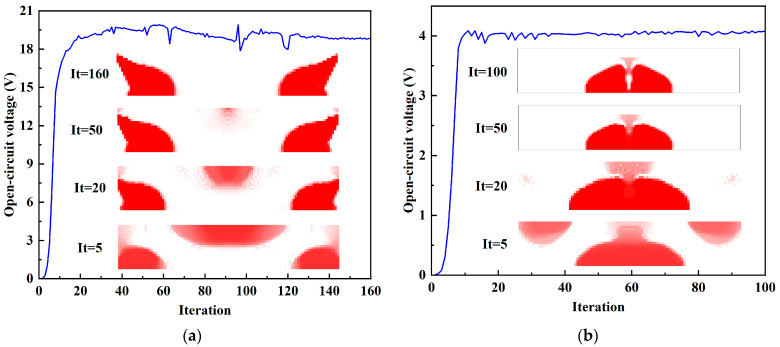
Iterative history and topological evolution for open-circuit voltage designs. (**a**) *f* = 4 kHz; (**b**) *f* = 16 kHz.

Figure 10 shows the iterative history and the topological evolution of the open-circuit voltage designs at 4 kHz and 16 kHz. The open-circuit voltage tends to converge after 40 iterations in Figure 10a. It can also be seen that some fluctuations appear in the convergence curve because a part of the piezoelectric materials distributes near the top edge, which can produce voltage oscillations. By the post-processing scheme with threshold, the final open-circuit voltage is 18.78 V, and the energy conversion efficiency is 12.02%. Figure 10b shows that the piezoelectric material tends to distribute towards the center of the structure at an excitation frequency of 16 kHz.

**Figure 11:**
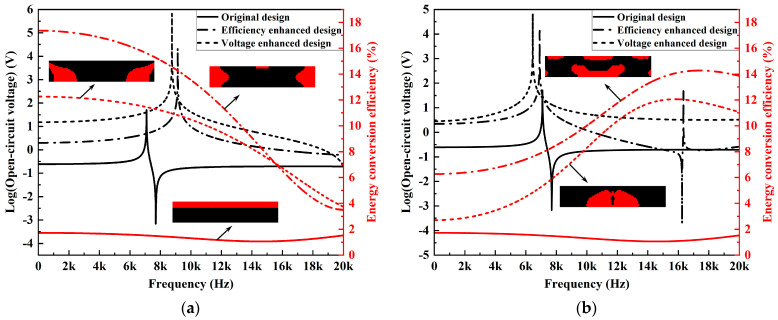
Frequency response results of the open-circuit voltage and energy conversion efficiency in different designs. (**a**) *f* = 4 kHz; (**b**) *f* = 16 kHz.

The frequency response results of the open-circuit voltage and energy conversion efficiency for the designs obtained under excitation frequencies of 4 kHz and 16 kHz are compared in Figure 11. The first two eigenfrequencies of the original design in Figure 2 are 7088 Hz and 16,121 Hz. As PZT-5A material has a comparable modulus to the substrate, the newly designed harvesters dynamically behave similarly to the original design with adjacent eigenfrequencies. Abrupt jumps in the frequency response curves can be observed at the resonance frequencies. For both excitation frequencies (4 kHz and 16 kHz), voltage-enhanced designs outperform the efficiency-enhanced designs in terms of the open-circuit voltages over almost the complete frequency range. Meanwhile, the comparison results show also that the enhanced designs for specific operation frequencies can, in fact, benefit the energy harvesting performance over a much broader working bandwidth.

## 5. Conclusions

This work proposed a topology optimization approach to achieve piezoelectric energy harvesters with enhanced energy conversion efficiency or open-circuit voltage. The piezoelectric material layout along the thickness direction was topologically optimized for the two considered design objectives. It can be concluded from the numerical results that: (i) for realizing a high energy conversion efficiency, the piezoelectric materials should be placed at higher stress/strain regions to generate more electricity energy; (ii) for realizing a high open-circuit voltage, the distribution of piezoelectric materials should be tuned to match the external excitation frequency to obtain larger electric power under constant electric resistance. Meanwhile, it can be inferred that the voltage cancellation issue can be evidently ameliorated by means of open-circuit enhancement designs. In the future, the design of PEHs circuits with varying resistance needs to be integrated into the piezoelectric dynamic system. Furthermore, the polarization directions and the manufacturability of piezoelectric materials will be considered to achieve high-efficient PEHs with a broader operational bandwidth.

## Figures and Tables

**Table 1:** The FEM analysis results of the energy conversion efficiency and open-circuit voltage.

Boundary Conditions	Frequency	Energy Conversion Efficiency	Open-Circuit Voltage
Without equipotential constraint	*f* = 4 kHz	4.84%	Not applicable
	*f* = 16 kHz	4.33%	Not applicable
With equipotential constraint	*f* = 4 kHz	1.66%	0.26 V
	*f* = 16 kHz	1.09%	0.19 V

**Table 2:** The optimized results of the open-circuit voltage enhanced designs.

Frequency	Energy Conversion Efficiency	Open-Circuit Voltage
*f* = 4 kHz	16.85%	2.46 V
*f* = 8 kHz	15.21%	8.91 V
*f* = 12 kHz	12.31%	0.51 V
*f* = 16 kHz	14.12%	0.13 V

**Table 3:** The optimized results of the open-circuit voltage enhanced designs.

Frequency	Voltage	Efficiency	1st Eigenfrequency	2nd Eigenfrequency
*f* = 4 kHz	18.78 V	12.02%	8755 Hz	17,344 Hz
*f* = 6 kHz	21.07 V	7.42%	7075 Hz	18,815 Hz
*f* = 10 kHz	41.78 V	13.53%	9188 Hz	18,966 Hz
*f* = 16 kHz	3.26 V	12.05%	6450 Hz	16,509 Hz

## Data Availability

The data presented in this study are available on request from the corresponding author.

## Appendix A

In terms of the design for energy conversion efficiency, the three penalization coefficients need to satisfy the following two conditions [27,28]:

(A1) $$\;2\times p_{u\phi }-\left(p_{uu}+p_{\phi \phi }\right)>0$$

(A2) $$\;p_{\phi \phi }-p_{uu}>0$$

where the first condition ensures a higher energy conversion efficiency along with the increase in piezoelectric material usage, while the second condition facilitates the convergence of the topology variables towards the upper and lower bounds. As commonly suggested, the three penalization coefficients are set as $p_{uu}=3$, $p_{u\phi }=6$ and $p_{\phi \phi }=4$. However, the second condition is not applicable when it comes to the design for open-circuit voltage. Numerical experiments show that the topology can only be converged provided that $p_{\phi \phi }<p_{uu}$. Hence, the penalization coefficients are empirically set as, and $p_{uu}=3$, $p_{u\phi }=6$ and $p_{\phi \phi }=1$ for open-circuit voltage designs. The resulting intermediate densities in the final design are enforced to the two bounds by a post-processing scheme with a threshold [41].
